# Observations on Particular Modes of Treatment Recommended to Assist Nature in Her Efforts during Parturition

**Published:** 1819-04

**Authors:** 


### For the London Medical and Physical Journal.

### *Observations on particular Modes of Treatment recommended to assist Nature in her Efforts during Parturition*; by F. T. H.

ANY interposition of art in the operations of nature under parturition must appear censurable, when urged as a general maxim to be acted upon in those cases which the united testimony of all ages has shown do not require any artificial aid whatever: by such cases I mean that immense majority of labours which the powers of nature alone are known to be fully adequate to complete, with the most perfect safety both to the mother and child. That a great deal has been done in every age to assist nature in her efforts during parturition, is well known; nor is it to be wondered at that, at the present day, in countries where fanaticism and ignorance hold the place of reason and philosophy, many absurd practices still prevail in the management of this important function. It is, however, the object of the present paper to maintain, in opposition to the practice recommended by the late Dr. Osborne, but at the same time with the greatest respect for that gentleman's high attainments, that the mode of interfering in natural labours, by *retarding* the birth of the child, is as unnecessary and as objectionable as *accelerating* the birth would be: first, because labour is in itself a natural healthy function, and requires no more artificial aid than the function of respiration does; secondly, because retarding the delivery can no more justly be said to prevent those accidents which now and then occur after the birth of the child, than the accelerating the delivery can be said not to be the cause of them.

With regard to the first objection, on the ground of labour being in itself a natural healthy function, and consequently requiring no assistance from art, little need be said, when it is recollected how many women have had their labours safely concluded without the aid of any attendant. In many of such cases, no doubt, the uterus had acted with such vigour as would have inclined a person of Dr. Osborne's opinion to have retarded the birth of the child, to have secured a more speedy and safe expulsion of the placenta. But I appeal to those gentlemen who have frequently come into the lying-in room after the child has been born, how often have they found the placenta lying in the vagina, and how seldom any disagreeable symptom from want of contraction of the uterus? It may, I allow, occasionally have occurred that, in women of irritable habits of body, who have suddenly been delivered of children, and no attendant near them, that, from anxiety and apprehension of danger, the uterus has either ceased to act altogether, or acted so irregularly, that the placenta has been retained: still such want of action, or irregularity of it, cannot fairly be attributable to the quick passage of the child through the os externum, when it is so well known, to every practitioner in midwifery, the powerful influence which passions of the mind exert over the action of the uterus.

With respect to the second objection to interfering in natural labour, by retarding delivery with a view to obviate the accidents that sometimes occur after the birth of the child, I have stated it to be as unjust to expect such a mode of practice to answer the end intended, as it would be to deny the possibility of the same accidents being produced by the opposite treatment. Now, it must be obvious that, either by forcibly dilating the external parts to facilitate the passage of the head through them, or after the head is born, dragging the body hastily through the os externum, the remainder of the labour,—viz. the expulsion of the placenta and subsequent contraction of the uterus,—may be rendered extremely tedious: for the external parts, being irritated by efforts used to dilate them, will sympathetically affect the uterus; and, if the recurrence of pain should not be entirely prevented, it will, in all probability, be of that kind which accompanies that partial and irregular contraction of the muscular fibres, known by the name of the hour-glass contraction of the uterus. The dragging the body hastily through the os externum after the exit of the head, will be likely to produce the same inconveniences, and even danger, where there is any disposition to flooding. But, though this is the opposite mode of conducting a natural labour to that recommended by Dr. Osborne, and now employed by many men of eminence, and which I allow to be not only erroneous but even sometimes dangerous, still I do not see that, on the present occasion, we can rationally found our practice on the maxim of Hippocrates, that "*τὰ ἐναντία τῶν ἐναντίων ἐϛὶν ἰάματα*;" and, consequently, believe that the retarding the birth must be right. On the contrary, I contend that the retarding the birth does often fail to secure a speedy contraction of the uterus, and is even sometimes productive of the very inconveniences which I have above stated precipitancy of conduct may give rise to. And can such an effect be wondered at, when it is considered that, by retarding the birth, we are opposing an unnatural resistance in the room of a natural one, which has been happily overcome, and hereby stimulating the uterus to fruitless and fatiguing efforts, which, if not carried to such extent as ultimately to impair the powers of the uterus, will, no doubt, if the efforts made to overcome the unnatural resistance be violent, dispose it to inflammation and severe after-pains. In cases even where the uterus has shown imperfect action, I do not see how offering resistance to the passage of the child can secure a more vigorous contraction after it is allowed to be expelled.

Dr. Clarke, of Dublin, has been in the habit of retarding the delivery in cases of imperfect action of the uterus; but he has also conjoined another sort of practice with this,—that of laying the hand upon the abdomen, and following the uterus in its contractions until the child is expelled. It is to the last practice, that of using the hand as a support to the uterus in these cases of weak action, that I attribute Dr. Clarke's success; for, as muscles evidently have their power increased by giving support to them whilst in action,—as, for instance, in the extremities, where the muscles are bound down and supported by strong fasciæ,—so this analogous support of the hand will enable the uterus to act with increased power. Still, provided we are able in this way to increase the powers of action in the uterus, I believe that no good effect can reasonably be expected from increasing the resistance to the child's birth.

The object of what has been stated above, is to endeavour to prevent the interposition of art in ordinary natural labour, and to recommend that, in those cases where we think it necessary to do something to increase the powers of the parts concerned in parturition, that we imitate, as far as possible, the means which we see nature herself employ in other parts of the body to attain the same end,—viz. an increase of the power of action. I shall conclude by observing, that it never can be necessary or justifiable, in those who ought merely to be the ministers of nature, to presume to ordain the moment when she should complete her office.

*London; Feb.* 10, 1819.

